# High Accuracy and Cost-Effective Fiber Optic Liquid Level Sensing System Based on Deep Neural Network

**DOI:** 10.3390/s23042360

**Published:** 2023-02-20

**Authors:** Erfan Dejband, Yibeltal Chanie Manie, Yu-Jie Deng, Mekuanint Agegnehu Bitew, Tan-Hsu Tan, Peng-Chun Peng

**Affiliations:** 1 Department of Electrical Engineering, National Taipei University of Technology, Taipei 10608, Taiwan; 2 Department of Electro-Optical Engineering, National Taipei University of Technology, Taipei 10608, Taiwan; 3 Faculty of Computing, Bahir Dar Institute of Technology, Bahir Dar University, Bahir Dar 26, Ethiopia

**Keywords:** liquid level sensing, fiber optic sensor, deep neural network

## Abstract

In this paper, a novel liquid level sensing system is proposed to enhance the capacity of the sensing system, as well as reduce the cost and increase the sensing accuracy. The proposed sensing system can monitor the liquid level of several points at the same time in the sensing unit. Additionally, for cost efficiency, the proposed system employs only one sensor at each spot and all the sensors are multiplexed. In multiplexed systems, when changing the liquid level inside the container, the float position is changed and leads to an overlap or cross-talk between two sensors. To solve this overlap problem and to accurately predict the liquid level of each container, we proposed a deep neural network (DNN) approach to properly identify the water level. The performance of the proposed DNN model is evaluated via two different scenarios and the result proves that the proposed DNN model can accurately predict the liquid level of each point. Furthermore, when comparing the DNN model with the conventional machine learning schemes, including random forest (RF) and support vector machines (SVM), the DNN model exhibits the best performance.

## 1. Introduction

Accurately measuring liquid levels is critical in various industrial applications, such as chemical processes, oil, and water reservoirs, fuel storage in transporting systems, and construction maintenance and monitoring [1]. To achieve this end, different types of sensors were proposed. Electrical sensors are one of the most common types of sensors [2]. However, their safety is an issue when used in hostile conditions, particularly in explosive or combustible environments [3]. Mechanic sensors, such as float sensors, are low-cost devices that meet safety requirements. However, their low resolution, vulnerability to mechanical damage, expensive maintenance costs, and higher system weight and volume, limited their applicability [3]. In circumstances that needed contactless liquid level sensing, radio frequency radar-based sensors were considered as a solution. Nevertheless, the polluted environment between the liquid and the sensor can easily cause inaccuracy in measurements [4].

Recently, fiber optic sensors (FOSs) were given priority over all other types of sensors [5,6,7]. The FOSs’ priority is based on their built-in characteristics and innate safety in terms of resistance and immunity to chemical corrosion and electromagnetic interference, and low power consumption. Furthermore, these sensors received much attention due to their small size, large bandwidth, high accuracy and resolution, and especially high multiplexing capacity. OFSs are classified as intrinsic or extrinsic, the former one of which uses the optical fiber as a liquid level sensor probe, enabling the optical signal to be measured and modulated while the latter only employs optical fiber as the signal transmission medium and an external modulation device as the sensor. Various FOSs configurations for monitoring liquid levels, such as long-period gratings (LPG), Fabry–Perot cavities, fiber Bragg gratings (FBG), and multimode interference were presented in recent years [8,9,10,11,12]. In [10], etched chirped fiber Bragg grating (CFBG) is proposed as a liquid level sensor. When the etched CFBG is submerged in liquid, its effective index changes, thus the phase and Bragg wavelength of the reflected spectrum will change. However, these schemes only concentrated on measuring the liquid level of a single spot or a single container instead of measuring multiple spots or containers simultaneously. Furthermore, to achieve higher accuracy in sensing a liquid level, several studies [8,11,13] use more than one sensor to measure the liquid level for one container or one spot. In [8], to interrogate several sensors, a machine learning method was used to predict the water levels by using nine FBG sensors. However, the prediction error still is more than 7 cm in some cases and more than 3 cm on average. Furthermore, although an increasing number of sensors may lead to an increase in accuracy, using numerous sensors for only one container or spot is not cost-effective, particularly in industries that need to measure a large number of containers simultaneously or at different points of a large reservoir. A different approach for measuring liquid level, such as measuring fluid level in non-stationary tanks, such as car fuel tanks, was proposed in [14], in which they used a single tube capacitive sensor. When there is dynamic slosh, the system determines the fluid level. Afterward, the sensor signal is processed with different methods, which have a maximum error of 8.7% for the distributed time delay neural network (DTDNN) and a maximum error of 0.11% for the backpropagation neural network. In [15], an IoT-compatible water level surveillance system was suggested, in which the surveillance system is made to make it easy to collect data. The technique, which is based on a convolutional neural network, yields to predict water levels that have an average error of 0.016 m. Additionally, in [16] a deep learning-based method for measuring water level based on YOLOv5s and the convolutional neural network was developed. The suggested technique extracts the water gauge area and all scale character areas from the original video images using YOLOv5s, then locates the location of the water surface line using image processing technology, and determines the elevation of the actual water level. The suggested method was validated with a video monitoring station and revealed that its systematic error is 7.7 mm. Despite their advantages, these methods face several challenges when the visual scenes are obscured or in environments where the deployment of surveillance systems is prohibited.

All the mentioned schemes only concentrated on measuring the liquid level of a single spot or a single container, and measuring the liquid level of multiple spots or containers at the same time was not investigated.

To increase the number of sensors in a system and to measure multiple spots or containers at the same time, one important method is multiplexing the sensors. The wavelength division multiplexing (WDM) method is one of the most common multiplexing techniques in fiber sensor systems to increase the number of sensors and monitor various parameters while reducing costs [17,18]. However, one drawback of a WDM system is the limited operating range of cascaded sensors, which limits the number of sensors that can be multiplexed in the sensor system. Furthermore, still sensing the liquid level of different containers or spots at the same time is a challenging issue due to the variety of structures that can be used as a liquid level sensor and the interrogation of the multiplexed sensor. Thus, it is essential to design a liquid level sensing system that utilizes a smaller number of sensors for each container or spot in comparison with previous studies and can multiplex them to be cost-effective in terms of using less equipment to interrogate the sensor’s response.

Hence, in this paper, we proposed a high-accuracy and cost-effective liquid level sensing system based on the intensity and wavelength division multiplexing (IWDM) method. To measure the liquid level of each spot, only one FBG sensor was used. To enhance the multiplexing capability in the liquid level sensing system, we used multiplex sensors in different spots by using the IWDM method. Thus, we increased the number of spots where the liquid level could be sensed, as well as achieved a system with lower operation and installation costs. The IWDM method can improve the sensor system’s multiplexing capacity and increase the number of sensors compared to typical WDM techniques [19]. In the IWDM systems, the overlapping of cascaded sensors generates cross-talk and makes it difficult to differentiate each sensor’s response according to environmental parameters. Accordingly, we use a deep neural network (DNN) to accurately predict the liquid level in multiple spots at the same time for a liquid level sensing system. The DNN approach is proposed rather than machine learning methods [8] that are employed to increase the accuracy of prediction. In the end, the DNN performance was compared with the conventional machine learning methods. In general, the contribution of this study is summarized as follows:

- To the best of our knowledge, we propose a liquid level sensing system for measuring the liquid level of multiple spots/containers at the same time for the first time;
- Since we propose a water level sensor that only consists of one FBG sensor for each spot, the sensing system is cost-effective;
- We propose DNN to predict the liquid level of each container in the liquid level sensing system, which is more accurate than conventional machine learning methods and our system’s accuracy is higher compared to earlier research that used AI to predict the water level.

The rest of this paper is organized as follows: Section 2 illustrates the experimental setup and sensor structures. In Section 3, the DNN model configuration is introduced, and finally, in Section 4, the results and comparisons of this paper are discussed.

## 2. Experimental Setup and Sensor Structure

**Figure 1:**
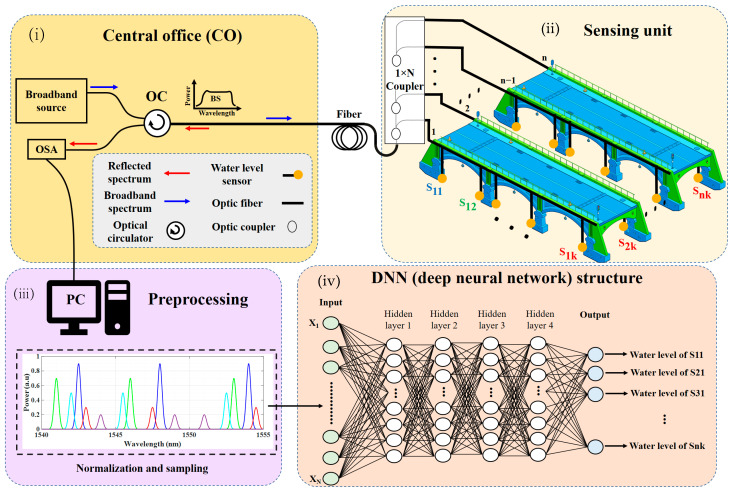
Scheme diagram of the proposed multiple-point liquid level sensing system, which consists of four main blocks: (i) central office, (ii) sensing unit which shows the IDWM liquid level sensing system, (iii) preprocessing unit, and (iv) deep neural network structure.

Figure 1 illustrates the schematic diagram of the proposed liquid level sensing system. The system consists of four parts: (i) The central office (CO), which is used to generate the broadband source that emits into the sensors and monitor the sensing system. The central office consists of a broadband source, optical spectrum analyzer (OSA), and optic circulator (OC). In the central office, the light generated from the broadband source is pumped into the liquid level sensors via OC and transmitted into the sensing unit using a fiber transmission channel. (ii) Sensing unit: in the sensing unit, the single-mode fiber (SMF) transmission channel is split into n rows by optical couplers. Each row has a different intensity ratio and consists of k sensors, which are labeled as S_n,k_ (n donates the row number and k donates the sensor number in the n^th^ row). Each sensor monitors the liquid level and generates a reflection spectrum dependent on the liquid level of that spot. Then, the reflected spectrum goes back to the central office through OC, and OSA is used to capture and record the reflected spectra of sensors at each stage of the liquid level. (iii) Preprocessing unit: we transfer recorded sensing data from OSA to a personal computer (PC) and then preprocess, normalize, and prepare to feed in the DNN. (iv) Deep neural network structure: includes the proposed DNN model that predicts the liquid level of each spot.

In this paper, the liquid level sensors are based on the FBG sensor. The FBGs are light-reflecting structures that transmit the broadband light spectrum while reflecting a semi-Gaussian spectrum in specific wavelengths. The Bragg wavelength (λB) is the center of the semi-Gaussian reflected spectrum, which is the peak wavelength of the reflected spectrum of a single sensor. The Bragg wavelength can be defined by the Bragg condition [20] for a uniform structure:

λB = 2η_eff_Λ (1)

**Figure 2:**
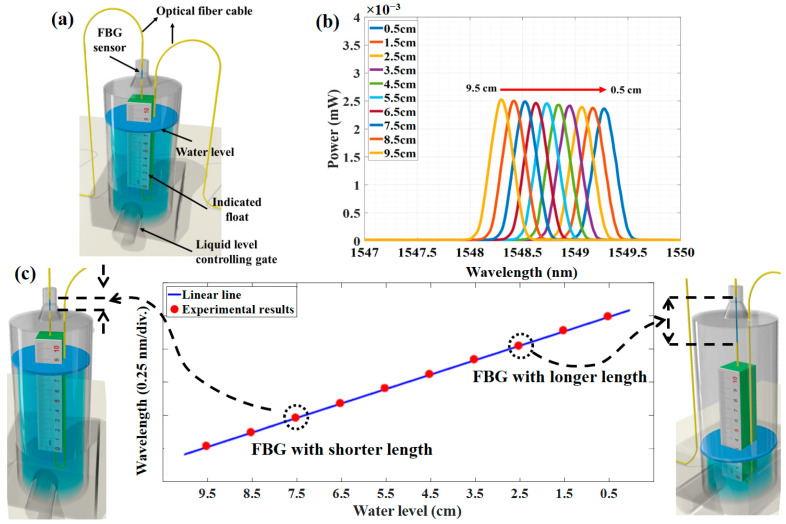
(**a**) The scheme of water level sensor structure that an FBG sensor connected to the indicated float. The water level can be controlled by the controlling gate. (**b**) The power versus wavelength of the reflected spectra of 10 different water level steps, and (**c**) the linear shift in wavelength when the water level change from 9.5 cm to 0.5 cm with the steps of 0.5 cm.

In Equation (1) the η_eff_ and Λ are the effective index and period of the grating, respectively. When a fiber is stressed, the center or Bragg wavelength of each FBG sensor shifts. Either compressive or tensile strain causes a change in fiber length and causes a variation in the period of the grating (Λ), which results in a shift in the Bragg wavelength. The following equation describes the relationship between wavelength change and strain [21]:

ΔλB = λB × (1 − ρε) × ε_m_ (2)

where ρε is the fiber’s photo-elastic coefficient and ε_m_ = ΔL/L is the mechanical strain and L is the length of FBG. Due to the dependence of the center wavelength on the strain, these structures can be employed as sensors that change in axial strain and will cause the Bragg wavelength shifts. To measure the liquid level, an FBG sensor was connected to the indicated float, as shown in Figure 2a. When the water level in the container changes, the float will move upward or downward, which causes axial strain and resulting a shift in the λB to the higher or lower wavelength.

To investigate the relation between water level changes and wavelength shift of the FBG sensor, the container was filled with water at room temperature. Then, we connected one sensor to the broadband source and applied a water level change in the container. Afterward, the OSA captured the reflected wavelength, while the water level in the container was changed by the controlled gate. As shown in Figure 2b, the center of the Gaussian reflected spectrum (λB) changes from 1548.25 nm to 1549.25 nm when the water level changes from 9.5 cm to 0.5 cm. Furthermore, to investigate the relation between the water level and the center of the Gaussian reflected spectrum, we change the water level with the steps of 0.5 cm from 9.5 cm to 0.5 cm (a total of 10 water level steps) and capture the reflected spectrum as well as the λB, as shown in Figure 2c. As shown in Figure 2c, the λB versus water level is shown, and according to this figure, it is possible to predict the water level by a linear transformation that converts the λB shifts to the water level changes.

Considering the scheme diagram of the optic fiber water level sensing system in Figure 1, when FBGs reflection spectra are assigned to the same wavelength range, varying degrees of overlapping might occur. Even so, a single peak may arise rather than several separate peaks when the reflected spectra are fully overlapping. Thus, finding the peak wavelength of each signal might be difficult. Therefore, a deep neural network (DNN) was proposed to predict the water level according to the Bragg wavelength shift of the sensors. To train the DNN model, the reflection spectra of each FBG were considered as R_i,j_, so the total reflected spectra that are recorded in OSA can be calculated as follows [22]:

(3) $$R_{\mathrm{total}}(\lambda )=\sum_{i=1}^{n}\sum_{j=1}^{k}R_{\left(i,j\right)}(\lambda ,\lambda B_{i,j})+N(\lambda )$$

In Equation (3), n is the number of rows, k is the number of sensors in each row, and we consider N(λ) in the system as a random white Gaussian noise. The R_i,j_ for each sensor can express as the following Equation [20]:

(4) $$R_{i,j}(\lambda ,\lambda B_{i,j})=I_{\mathrm{peak},i}\times \exp\left[-4\ln2\times {\left(\frac{\lambda -\lambda B_{i,j}}{\Delta \lambda_{\mathrm{FWHM}}}\right)}^{2}\right]$$

where I_peak,i_ is the peak reflectivity in the i^th^ row and Δλ_FWHM_ is the full width at half maximum intensity of the FBG sensor. The λB_i,j_ is the center wavelength of FBG_i,j_ that depends on its water level. In industrial projects, the sensor network could contain numerous rows and sensors in each row; however, in this paper, we focus on two-row (n = 2) and one sensor in each row (S11 and S21). Herein, the training dataset is generated using the aforementioned numerical methods with added noise. The model is then trained and validated using training data, and then the performance is tested using real experimental data. We collected real experimental data using two different scenarios. It is worth mentioning that the smoothing function of the optical spectrum analyzer (OSA) is applied during the experimental measurements to reduce noise and enhance the performance of the system.

## 3. DNN Model Configuration

Separating numerous overlapping spectra and predicting water level can be thought of as a regression problem, and a DNN is proposed to solve this problem. Recently, the DNN achieved cutting-edge performance in a variety of domains, including image recognition [23], 6G communication [24], recommendation systems [25], and visual art processing [26]. A DNN model consists of three main layers: input, hidden, and output. In a fully connected DNN, each neuron is connected to all neurons in the subsequent layer. Prediction values are obtained from the output layer after the hidden layers processed the data and extracted the features from the input layer. Each hidden layer neuron has an activation function that takes the weighted sum of nodes as input and transforms it into valid values. This activation function is used to calculate predicted values and to derive the weighted total of neurons.

In this study, we consider the DNN that contains L layers, and each layer has N[L] neuron, where L = 1, 2, 3, 4, 5 (four hidden layers and one output layer, as shown in Figure 1 in the DNN structure part). Since two sensors are investigated in the experimental setup, the N [5] = 2, which means there are two outputs. In each layer, the biases are represented by the b[L], which are (1 × N[L]) vectors. The (i × j) weight matrix for the L^th^ layer is represented by w_ij_[L], in which the i^th^ and j^th^ element of the matrix is the weight between the i^th^ neuron in the previous layer and the j^th^ neuron in the current layer. In each neuron, the activation function roughly applies the nonlinearity’s effect in the hidden layers. Given that all of the DNN’s weights and biases were obtained through forward propagation (FP) training, the total composite relationship between input and output is represented by Equation (5) [27]:

(5) $$\begin{array}{l} Y=f(f(f(f(X^{T}\times w[1]+b[1])\times w[2]+b[2])\times \\ \;w[3]+b[3])\times w[4]+b[4])\times w[5]+b[5] \end{array}$$

where Y is a 2 × 1 vector ([y_1_, y_2_]), f (.) is the activation function, and w[.] and b[.] are the weight matrix and bias of each layer, respectively.

Herein, the exponential linear unit (ELU) [28] activation function was considered for all layers, which are defined in Equation (6).

ELU(x) = max(0,x)+ min(0,α(e^x^ − 1)) (6)

The α is the ELU hyperparameter and determines the point at which a negative input ELU saturates. This activation function was chosen based on two reasons. Firstly, it was observed that in the hidden layers, the negative values will be produced as the weights and biases are updated; although, in the input layer, all values are positive. Therefore, to prevent data loss, ELU is a superior choice. Secondly, since we encounter a regression problem, it is recommended to use activation functions, such as ReLU, PReLU, SELU, and ELU for the output layer, and in this case, the ELU activation function shows better preference.

During each training session, the output layer forecast values and repeatedly modifies the weights. Backpropagation (BP) is used in this modification of the weights from the output layer to the input layer until the loss function reaches its minimum value. According to the type of problem, different loss functions were suggested [29]. In the regression circumstances where it is expected that the predicted outputs are real-number values, mean squared error (MSE) is a common loss function, which is defined as Equation (7) [30].

(7) $$\mathrm{Loss}=\frac{1}{n}\sum_{i=1}^{n}{\left(y_{i}-y{{}^{\prime }}_{i}\right)}^{2}$$

where n is the number of output layer neurons, and y_i_ and y’_i_ stand for the ith output’s real value and ith output’s predicted values, respectively. Afterward, the weight is modified according to Equation (8), and the next weight is equal to the difference between the previous weight and the partial derivative of the loss function [26].

(8) $$w_{\mathrm{ij}}^{t+1}=w_{\mathrm{ij}}^{t}-\eta \frac{\partial \mathrm{Loss}}{\partial w_{\mathrm{ij}}^{t}}$$

where the w^t^_ij_ donates the weight between the ith neuron in the previous layer and the j^th^ neuron in the next layer, t is the t^th^ iteration, and η is the learning rate.

Although the DNN model was used to predict the liquid level, it is crucial to predict the λB_i,j_ first. Afterward, as illustrated in Figure 2c, it is possible to convert λB_i,j_ to liquid level by a linear transform. To collect the dataset for training the DNN model and then to predict the liquid level, the OSA spectrum was generated through the theoretical method according to Equations (3) and (4). It is recommended to train the proposed DNN model by using sensor data having distinct intensity and varied reflection spectra to improve the model learning capacity. Furthermore, it helps increase measuring accuracy, as well as the versatility and adaptability of the proposed model. Therefore, we consider different peak powers of each sensor, respectively, and Δλ_FWHM_ = 0.25 nm for all sensors. Another important parameter for generating the dataset is the Bragg wavelength of sensors (λB_1,1_ and λB_2,1_), which was considered 50 different λB for each sensor in its operation bandwidth (1547 nm to 1550 nm). As a result, 12,500 different spectrum samples were produced and 100 samples were separated for testing. Then, from the remaining samples, 10% were chosen for validation. The training dataset (i.e., captured reflected spectrum) is preprocessed and normalized between 1 and 0.

Regarding the DNN input being the reflected spectrum, it is possible to consider different numbers of neurons for the input layer according to the number of sample points. Usually, the number of sample points is limited by the instruments’ sampling accuracy. Although it is possible to increase the sample points by different interpolation methods, in some cases it reduces intentionally to reduce the system complexity and computation time. According to this trade-off between accuracy and system complexity, we consider 1001 neurons for the input layer, which bring us adequate accuracy with appropriate complexity. Resultingly, the OSA simulated spectrum (R_total_(λ)) was sampled with 1001 points of wavelength (λ). Whereas our operation bandwidth is 1547 nm to 1550 nm, the first wavelength sample will be λ_1_ = 1547 nm and the last wavelength sample will be λ_1001_ = 1550 nm by using a wavelength shift (Δλ) of 0.003 nm at each step. On the other hand, the number of neurons in the output layer depends on the number of sensors in the sensing system, which in our case is two and labeled as the water level (WL_1,1_ and WL_2,1_) in the training data set. Thereby, each dataset consists of 1001 input features and two outputs denoted as {[R_total_(λ_1_), …, R_total_(λ_1001_)], [WL_1,1_, WL_2,1_]}.

**Figure 3:**
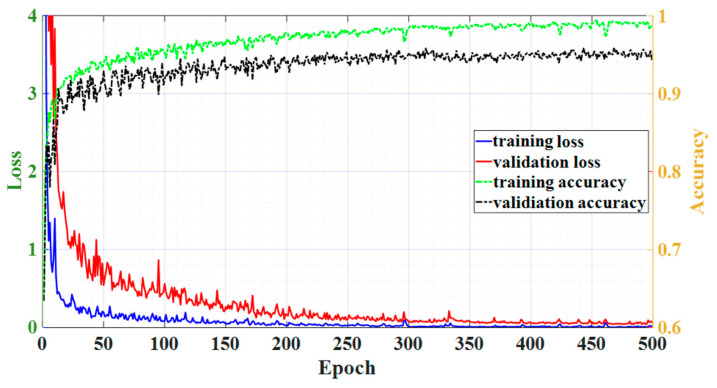
The DNN model performance in terms of loss and accuracy for training and validation dataset.

The DNN performance for the training and validation dataset is plotted in Figure 3. In Figure 3, the blue and red solid lines show the loss on the left axis for training and validation data, respectively, while the dashed green and black lines show the accuracy on the right axis. As shown in the figure, the training loss and validation loss converge at epoch 500 and it achieves a good performance at this epoch number. The optimal DNN parameters, including the learning factor, learning algorithm, and the number of neurons, are shown in Table 1. The number of neurons Є {32, 64, 128, …, 1024}, the number of hidden layers Є {2, 3, 4, …, 8}, and optimizer type Є {Adam, Adamax, RMSprop, SGD} values in Table 1 are derived by random search method, while other hyperparameters are chosen empirically.

## 4. Result and Discussion

As shown in Figure 1, the simplified experimental setup of the proposed liquid level sensing system is carried out by using two water level sensors (S11 and S21). These sensor models are CP-9000 and manufactured by Citpo Technologies Inc. Furthermore, the two sensors have identical operating wavelengths. To prove the reliability and stability of our proposed deep learning algorithm, two scenarios were considered. The first scenario is when both sensors’ liquid levels change simultaneously (S11 liquid level changes from 9.5 cm to 0.5 cm and S21 liquid level changes from 0.5 cm to 9.5 cm). While in the second scenario, the first sensor (S11) liquid level changes from 0.5 cm to 9.5 cm, and the second (S21) liquid level is fixed and set to 5 cm. We fill both sensors with water at room temperature for both experiments.

### 4.1. First Scenario: When Both S11 and S21 Sensors’ Liquid Levels Change Simultaneously

In this scenario to change the water level of sensors simultaneously, the sensors’ liquid level controlling gates are connected by a pipe. Therefore, by increasing the height of one sensor, the water flows to the other one. To change the height of the sensors, we use blocks with a height of 2 cm. It should be noted that adding a block with a height of 2 cm to a sensor will cause the water level to reduce by 1 cm in the sensor, while in another one, the water level will increase by 1 cm. In this scenario, 10 different water level steps were completed, in which the liquid level changed by 1 cm in each step. For each water level step, we allowed the water to stabilize before measuring the reflected spectrum, then the reflected spectrum was captured using OSA. We set the OSA sampling resolution and number of sampling points to 3 pm and 1001, respectively.

**Figure 4:**
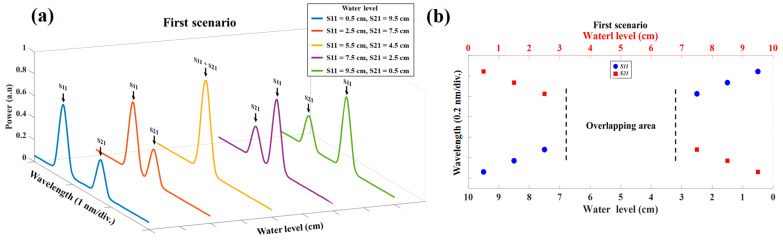
(**a**) The experimental result of the reflected spectrum of two water level sensors in five different water level steps of the first scenario. (**b**) The unmeasurable overlapping gap due to the overlap of two sensor spectra in the first scenario.

The experimental result of the reflected spectrum for five different water level steps for the first scenario when the water level in sensor S11 is 0.5 cm, 2.5 cm, 5.5 cm, 7.5 cm, and 9.5 cm is shown in Figure 4a. In this figure, the fully overlapping spectra can be observed in the third plot, which is impossible to measure the sensing signals of each sensor. Figure 4b depicts the water level of S11 and S21 versus their Bragg wavelength when the water level rises from 0.5 cm to 9.5 cm in the S21 but falls from 9.5 cm to 0.5 cm in the S11, which shifts in a different direction. The blue circles show the Bragg wavelength of S11 and the red squares are the Bragg wavelength of S21 for 10 different water level steps. As shown in the figure, the unmeasurable gap (overlapping area) occurred between the S11 and S21 sensors when the water level of the S11 sensor was in the range of 6.5 cm–3.5 cm. Since in this scenario, the Bragg wavelength of S11 and S21 shift in the opposite direction, the overlapping area is narrow.

### 4.2. Second Scenario: When Only the S11 Sensor Liquid Level Changes

In this scenario, the first sensor’s (S11) controlling gate is connected to a tank, while the second sensor’s (S21) controlling gate is closed. Then, the S11 and S21 sensors are filled with water to reach the 9.5 cm and 5cm of water levels, respectively. To reduce the water level in the S11, a similar method as mentioned in the first scenario is applied. Hence, the water level reduces from 9.5 cm to 0.5 cm by the steps of 1 cm in the S11, while during all these water level steps, the S21 water level remained at 5 cm. A similar setting for OSA as the first scenario is considered.

**Figure 5:**
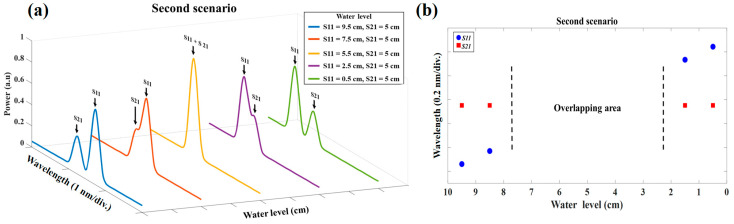
(**a**) The experimental result of the reflected spectrum of two water level sensors in 5 different water level steps of the second scenario. (**b**) The unmeasurable overlapping gap due to the overlap of two sensor spectra in the second scenario.

To compare this scenario with the former one, the experimental result of the reflected spectrum for five different water level steps is depicted in Figure 5a with the same amount of water in the S11 (the water level in S11 is 9.5 cm, 7.5 cm, 5.5 cm, 2.5 cm, and 0.5 cm). The first, second, and third plots in this diagram show the non-overlapping, partially overlapping, and entirely overlapping regions, respectively. As is shown in Figure 5a, in this scenario, it is much more difficult to measure the sensing signals of each sensor when the reflection spectra of two sensors are partially overlapping or completely overlapping. Figure 5b shows the water level for S11 and S21 versus their Bragg wavelength when the water level in the S11 decreases from 9.5 cm to 0.5 cm and when the S21 water level is 5 cm. The S11 and S21 Bragg wavelength for 10 different water level steps is shown with blue circles and red squares, respectively. As shown in Figure 5b, the unmeasurable gap (overlapping area) occurred between the S11 and S12 sensors when the water level of the S11 sensor was in the range between 7.5 cm and 2.5 cm. In this scenario, two important reasons caused the overlapping area to be bigger than the former one: firstly, the constant Bragg wavelength of S21 in all experiments, and secondly, its position, which is in the middle of the sensor’s operation bandwidth. Consequently, predicting the water level in this scenario is more challenging rather than in the first scenario.

### 4.3. Performance Evaluation

**Figure 6:**
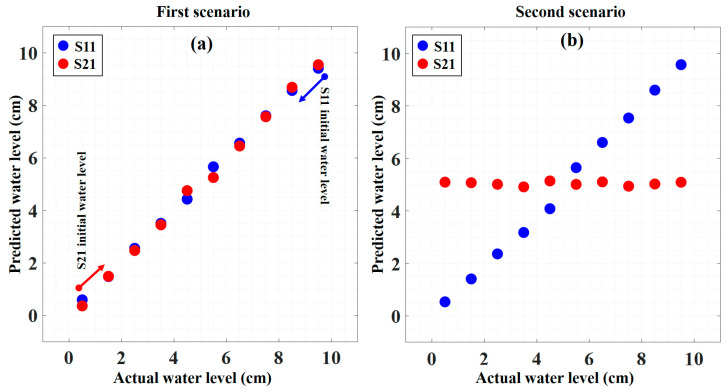
The predicted water level versus the actual water level for the (**a**) first scenario and (**b**) second scenario.

The performance of our proposed deep neural network model for the IWDM water level sensing system is discussed in this section. Therefore, we used the unseen test data of experimental results from the first and second scenarios to test the well-trained DNN model. The test data consists of 10 water level steps for each scenario. In the preprocessing part, the captured reflected spectrum normalized between 1 and 0. It should be noted that since in the OSA setting the number of sample points was considered to be 1001, there is no need for up/down sampling. Hence the experimental test data after normalization can be directly fed to the DNN. The performance of the proposed model for the first and second scenarios is shown in Figure 6 by plotting the predicted water level versus the actual one. For both scenarios, the blue and red circles show the S11 and S21 predicted water levels, respectively.

To evaluate the performance of our DNN model on the experimental test data, we use the mean absolute error (MAE) evaluation method, which is defined as follows:

(9) $$\mathrm{MAE}=\frac{1}{n}\sum_{n=1}^{n}\left|n_{n}-y{{}^{\prime }}_{i}\right|$$

where the y_i_ and y’_i_ are the actual water level and the predicted water level, respectively, and n is the number of predictions for each scenario as far as we have two sensors.

**Figure 7:**
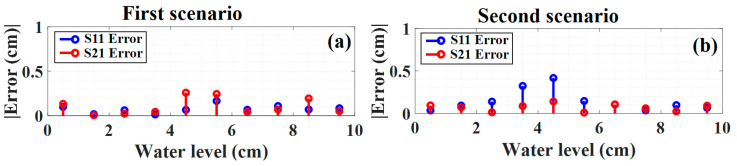
The water level prediction error versus the water level for the (**a**) first scenario and (**b**) second scenario.

In Figure 7 the water level prediction error of both sensors versus the water level for each scenario is depicted. The blue and red colors show the error for the S11 and S21, respectively. As mentioned before, the bigger overlapping area in the second scenario makes prediction more challenging, thus the error in the second scenario increases slightly. The MAE for the S11 and S21 sensors for the first scenario is 0.0748 cm and 0.1063 cm, respectively. Hence, the total MAE for the first scenario can be calculated by averaging sensor errors, which for the first scenario is 0.0906 cm. For the second scenario, the MAE for the S11 and S21 for the first scenario are 0.1405 cm and 0.0693 cm, and the total MAE for this scenario is 0.1074 cm.

Based on the result of these two scenarios, our proposed model can predict the water level of each sensor even from the completely overlapping spectra. Furthermore, we compare and contrast our proposed DNN approach with two conventional machine learning methods, such as random forest (RF) and support vector machines (SVM) to prove the reliability of our proposed model for water level prediction. We used the same training and testing data under the same PC environment. To train the random forest (RF) and support vector machines (SVM), the random hyperparameter grid method is considered to find the parameters. Table 2 and Table 3 show the optimal parameters after using the random search method for tuning the hyperparameters for training the random forest and SVM models, respectively.

**Figure 8:**
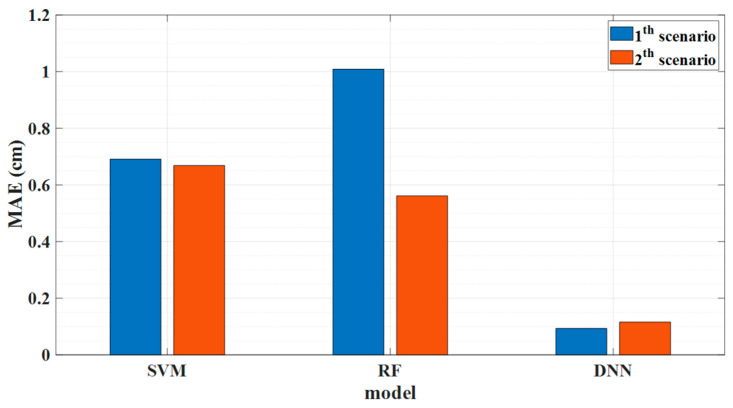
Depiction of the mean absolute error (MAE) of experimental results for the support vector machine (SVM), random forest (RF), and deep neural network (DNN) algorithm.

Figure 8 shows the water level prediction performance of SVM, RF, and DNN in terms of MAE for both scenarios. In contrast to RF models, which may not perform as well in complicated datasets, SVR models can simulate complex non-linear connections between the input features and the target variable. As a result, the MAE of SVM generally outperforms RF. Additionally, SVR models are more robust than RF models, so SVM performance in both scenarios is nearly identical, in contrast to RF, which is not the case. Considering that DNN models can handle high-dimensional data more effectively, which can lead to improved performance, compared to RF and SVM models, the MAE of water level prediction manifests superior performance for both scenarios. Additionally, it is worth noting that the training time for DNN is much shorter than the conventional machine learning method for the same amount of dataset.

**Figure 9:**
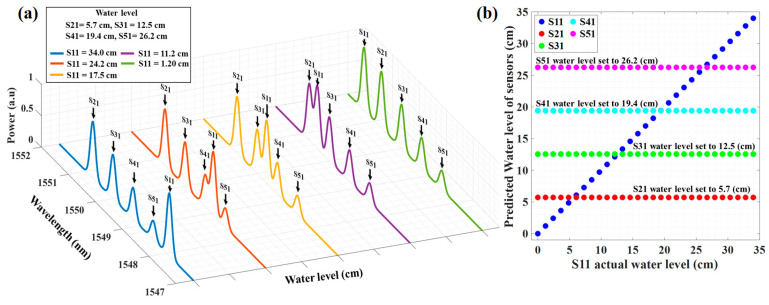
(**a**) The simulation results of reflected spectra of five water level sensors in five different water level steps, and (**b**) the predicted water level versus the actual water level of five sensors.

Additionally, to affirm the competence and capability of the proposed system, a simulation of the system with five sensors was conducted. All five sensors (S11–S51) are considered to work in the same operating wavelength range (1547 nm to 1550 nm). The water level of S11 changed from 0 to 34 cm, while the water level of other sensors (S21–S51) is set to be 5.7, 12.5, 19.4, and 26.2 cm, respectively. Thus, the reflected spectra of five FBGs (i.e., training dataset) at different water level steps are collected by simulation using Equation (4). The computer-simulated reflected spectrum data of five sensors are displayed in Figure 9a. As shown in the figure, it can be seen some of the spectra of two FBGs are partially or fully overlapped. To train the DNN, we generated a dataset that consists of five sensors’ reflected spectra. Afterward, the DNN model is trained with the dataset, and to prove the performance of the well-trained model, we use unseen testing data of five sensors’ reflected spectra.

As it is depicted in Figure 9, even as the number of sensors increases, the proposed DNN model can reliably measure the water level of each container. This indicates that we can accurately predict the Bragg wavelengths of each sensor and the water level of each container, even though the reflected spectra of two or more FBG sensors are partially or fully overlapped. Furthermore, our system performs better in terms of the number of sensors and the accuracy of the water level prediction in comparison with [8]. Additionally, in terms of multiplexing the sensor and interpreting the sensors’ response in comparison with [13], the proposed system does not need to reserve two channels and the accuracy of water level prediction is not dependent on the number of sensors.

## 5. Conclusions

In this paper, a novel liquid level sensing system is proposed to enhance the capacity of the sensing system and reduce the cost. The proposed sensing system can monitor the liquid level of several points at the same time, which is cost-effective. Moreover, to overcome the overlapping challenge in IWDM-based sensor networks, we propose a deep neural network (DNN) structure. The proposed model first predicts the Bragg wavelength and then predicts the water level. Two different scenarios were considered to investigate DNN performance. Hence, the mean absolute error is used as a metric to evaluate the DNN model water level prediction performance. The result proves that the proposed DNN model accurately predicts the liquid level of each sensor. We achieve the average MAE of 0.09 cm for the first scenario with minimum and maximum errors of 0.01 cm and 0.26 cm. Additionally, for the second scenario, we get the average MAE of 0.1 cm with minimum and maximum errors of 0.01 cm and 0.4 cm. In the end, when comparing our proposed DNN model with other conventional machine learning algorithms, such as random forest (RF) and support vector machines (SVM), the proposed DNN model exhibits the best performance.

## Figures and Tables

**Table 1:** The deep neural network (DNN) parameters.

DNN Parameters	Value
Number of neurons in the input layer	1001
Number of hidden layers	4
Number of neurons in each layer	350
Activation function	ELU
ELU hyperparameter (α)	1
Optimizer algorithm	Adam
Learning rate (η)	0.001
Batch size	250
Number of epochs	500

**Table 2:** The random forest (RF) model parameters.

Random Forest Parameters	Value
The number of trees in the forest	502
The maximum depth of the tree	None
The minimum number of samples required to split an internal node	3
The minimum number of samples required to be at a leaf node	1

**Table 3:** The Support-vector machines (SVM) model parameters.

SVM Parameters	Value
The kernel type	‘rbf’
Epsilon	0.0001
Kernel coefficient for ‘rbf’ (gamma)	0.01
Tolerance for stopping criterion	0.0001

## Data Availability

Not applicable.
